# Genetic Determinants of Infectious Diseases in the Qatari Population

**DOI:** 10.3390/epidemiologia7040110

**Published:** 2026-08-14

**Authors:** Maria K. Smatti, Yasser A. Al-Sarraj, Omar Albagha, Hadi M. Yassine

**Affiliations:** 1 Biomedical Research Center, QU Health, Qatar University, Doha P.O. Box 2713, Qatar; 2 College of Health and Life Sciences (CHLS), Hamad Bin Khalifa University (HBKU), Doha P.O. Box 34110, Qatar; 3 Qatar Precision Health Institute (QPHI), Qatar Foundation, Ar-Rayyan P.O. Box 5825, Qatar; 4 Diabetes Research Center (DRC), Qatar Biomedical Research Institute (QBRI), Hamad Bin Khalifa University (HBKU), Doha P.O. Box 34110, Qatar

**Keywords:** host variants, infectious diseases, SNPs, Qatari genomes, susceptibility

## Abstract

Background and Objectives: Human populations show striking phenotypic disparities in traits and diseases, including responses to infectious diseases (IDs). The impact of host genetic diversity on infection susceptibility and outcomes is increasingly recognized, yet remains largely unknown in Qatar. Here, we explored the distribution of infection-related host genetic variants in Qatar. Materials and Methods: Infection-related genetic variants from the GWAS Catalog were analyzed in 6047 Qatari whole genomes from the Qatar Genome Program (QGP) and compared with populations from the 1000 Genomes Project (1000G). Results: Out of 272,610 GWAS Catalog associations, 1086 were related to ID susceptibility, resistance, severity, progression, clearance, response to treatment, or vaccination, and hence included in the subsequent analysis. A significant heterogeneity in the allelic frequencies (AFs) between Qatari (*n* = 6047) and the 1000G populations (*n* = 2504) was observed. The QGP cohort carries significantly lower AFs of most risk variants associated with susceptibility to tuberculosis, malaria, hepatitis, diarrheal disease, and shingles (up to 222-fold, *p* < 0.0001). Contrarily, an enrichment in the AFs of variants that increase the risk of chickenpox, plantar warts, pneumonia, urinary tract infections (UTIs), and leprosy was observed among Qatari individuals, yet with much smaller-fold differences (≤5-fold, *p* < 0.0001). In addition, most severity/chronicity-related variants were considerably less prevalent in the Qatari population (up to 20.5-fold). Analysis of variants associated with antibody response revealed a distinct genetic distribution, especially in Epstein–Barr virus (EBV) and *Chlamydia pneumoniae* infections (27.8- and 3-fold, respectively). Moreover, a variable inter-population allelic distribution was observed in SNPs related to measles, mumps, and rubella (MMR), smallpox, and hepatitis B virus (HBV) vaccine response, as well as variants linked to viral clearance, viral load, virus-induced progression to cancer, and response to treatment. Conclusions: These findings reveal substantial differences in pathogen-associated host variants in a diverse Qatari cohort and highlight the need for follow-up validation and future GWAS discovery efforts.

## 1. Introduction

Despite all the efforts to decrease the global burden of IDs, the world repeatedly faces recurrent outbreaks/pandemics caused by emerging or re-emerging pathogens. In fact, although the overall number of people dying from infectious diseases is declining each year, the number of newly identified viruses, such as acute respiratory syndrome coronaviruses (SARS-CoV)-1 and 2 and human immunodeficiency virus (HIV), has nearly quadrupled over the past century [1]. Moreover, the world has witnessed triple the number of outbreaks per year since 1980 [2]. The WHO estimates that IDs will lead to 13 million deaths by 2050 [3]. Additionally, with the threat of widespread antimicrobial resistance (AMR), some research groups have warned that by 2050, infectious diseases will possibly surpass cardiovascular diseases as the world’s number one killer and lead to healthcare costs of at least $100 trillion [4]. In the Eastern Mediterranean Region (EMR), which consists of countries in North Africa and the Middle East, along with Afghanistan, Cyprus, and Pakistan, infectious diseases remain a leading cause of morbidity and mortality, accounting for one-third of all death and illnesses [5]. Countries in this region have been experiencing an alarming number of emerging and re-emerging IDs, including SARS-CoV-2, Middle East Respiratory Syndrome (MERS), acute watery diarrhea, avian influenza, cholera, chikungunya, chickenpox (varicella), and travel-associated Legionnaire’s disease [6]. As a part of the EMR, with the high number of multi-national expatriate influx to the country, Qatar continues to face a considerable challenge in infection control. According to the Qatar Ministry of Public Health (MoPH), communicable diseases account for around 8% of all deaths in Qatar, negatively affecting the life quality of residents and creating an overwhelming concern for Qatar’s healthcare system [7].

It is now well evidenced that the susceptibility, pathology, and subsequent morbidity and mortality during infections are shaped by a complex interaction of pathogen, environmental, and host genetic factors [8]. Previous reports highlighted the possible role of heritable factors in shaping the inter-individual and inter-population differences in clinical outcomes [9]. Historically, twin studies have been the primary source of knowledge in evaluating and supporting this hypothesis. In the last decade, however, the paradigm shift to genome-wide association studies (GWASs) has provided additional, larger-scale analysis of host genetics in infectious diseases [10]. Since the first GWAS on infectious disease (HIV) in 2007, numerous host variants have been identified and linked to infection acquisition or resistance. Nevertheless, such genomic studies remain inadequate due to the limitations in population inclusion and the number of studied infections. In fact, single-ancestry European cohorts represent 81% of all GWASs, while Asians represent 14%, which collectively account for 95% of all GWAS populations. This evidently indicates the underrepresentation of other ancestries in genomic studies on different phenotypes, including IDs. Importantly, understanding population genetics that contribute to infection spread could have an innovative implication for infection control beyond the traditional approaches.

Disparities in the genetic predisposition to infectious diseases in the Arab population generally and the Qatari population specifically remain largely undefined. In Qatar, the launch of the Qatar Genome Program has paved the way for population genomic studies that investigate genetic variation in disease-causing loci for a wide range of population-relevant traits. In this study, we utilized the first-phase release of the QGP, which consisted of 6218 whole genomes, to examine a large set of infectious disease-related host genetic variants. This study aimed to provide preliminary insights into the genetics of infectious diseases in Qatari compared to other populations.

## 2. Methods

### 2.1. Study Subjects

The study population included a subset (*n* = 6218) of Qatar Biobank (QBB) participants whose genomes were sequenced as part of the first phase of the Qatar Genome Program (QGP). The cohort’s full demographic description has been previously described [11]. All subjects are adult (age ≥ 18 years) Qatari nationals. The mean age of enrolled subjects was 40.5 years (SD ± 12).

### 2.2. Genomic Data

Whole-genome sequences were obtained. Sequencing read data were previously produced by Illumina HiSeq X Ten sequencers (Illumina, Inc., San Diego, CA, USA) and converted from the native BCL format to paired-end FASTQ format using bcl2fastq (v2.20). Fastqc (v0.11.2) was used to assess the quality of the raw data. Data passing quality control was then aligned to the reference genome sequence (build GRCh37 (hs37d5)) using the bwa-kit aligner [v7.12]. Variant calling and the subsequent annotation of the resulting VCF were performed by GATK haplotype caller [v3.3] and snpeff [v4.1b] (databases dbsnp v138 and dbNSFP v2.9), respectively. The genetic variant data was then converted to PLINK file format using PLINK-1.9 [12]. The resulting data was then subjected to standardized quality assurance and quality control (QA/QC) processes to generate high quality and confidence on both SNP and sample levels, as previously described [13]. Briefly, we removed variants with genotype call rate < 90%, and Hardy–Weinberg *p*-value < 1 × 10^−6^. In addition, samples with excess heterozygosity (*n* = 8), duplicates (*n* = 10), call rate < 95% (*n* = 1), gender ambiguity (*n* = 65), and population outlier (*n* = 87) were removed. The final data file used for allele frequency analysis included 6047 subjects.

### 2.3. SNP Selection

We used the NHGRI-EBI Catalog of human genome-wide association studies (GWAS catalog: https://www.ebi.ac.uk/gwas/, accessed on 3 July 2021) to extract and summarize variants that were reported to be associated with any infectious disease. Initially, we downloaded the summary tables covering all the associations in the GWAS catalog. This included 272,610 associations, of which 202,813 were statistically significant according to the standard GWAS *p*-value threshold (≤5 × 10^−8^). Next, a filtration step was carried out to select associations that are related to infectious disease susceptibility, resistance, severity, progression, clearance, response to treatment, or vaccination. This resulted in 1086 associations from 219 studies, of which 883 were unique SNPs that were included in the subsequent analysis.

### 2.4. Data Analysis

We compared the allele frequencies between the Qatari population and the 1000G populations (AFR: African; AMR: American; EAS: Eastern Asian; EUR: European; SAS: South Asian). Pearson’s Chi2 test was used to investigate significant differences. To account for multiple comparisons, *p*-values were adjusted using the Benjamini–Hochberg false discovery rate (FDR) method. Statistical significance is indicated as follows: FDR-adjusted *p* < 0.05 (*), FDR-adjusted *p* < 0.01 (**), FDR-adjusted *p* < 0.001 (***), and FDR-adjusted *p* < 0.0001 (****). ns: not significant. GraphPad Prism 7 (GraphPad Software, LLC, Boston, MA, USA) was used for analysis and graph plotting.

## 3. Results

### 3.1. The Overall Genetic Differences in Variants Associated with IDs

**Figure 1:**
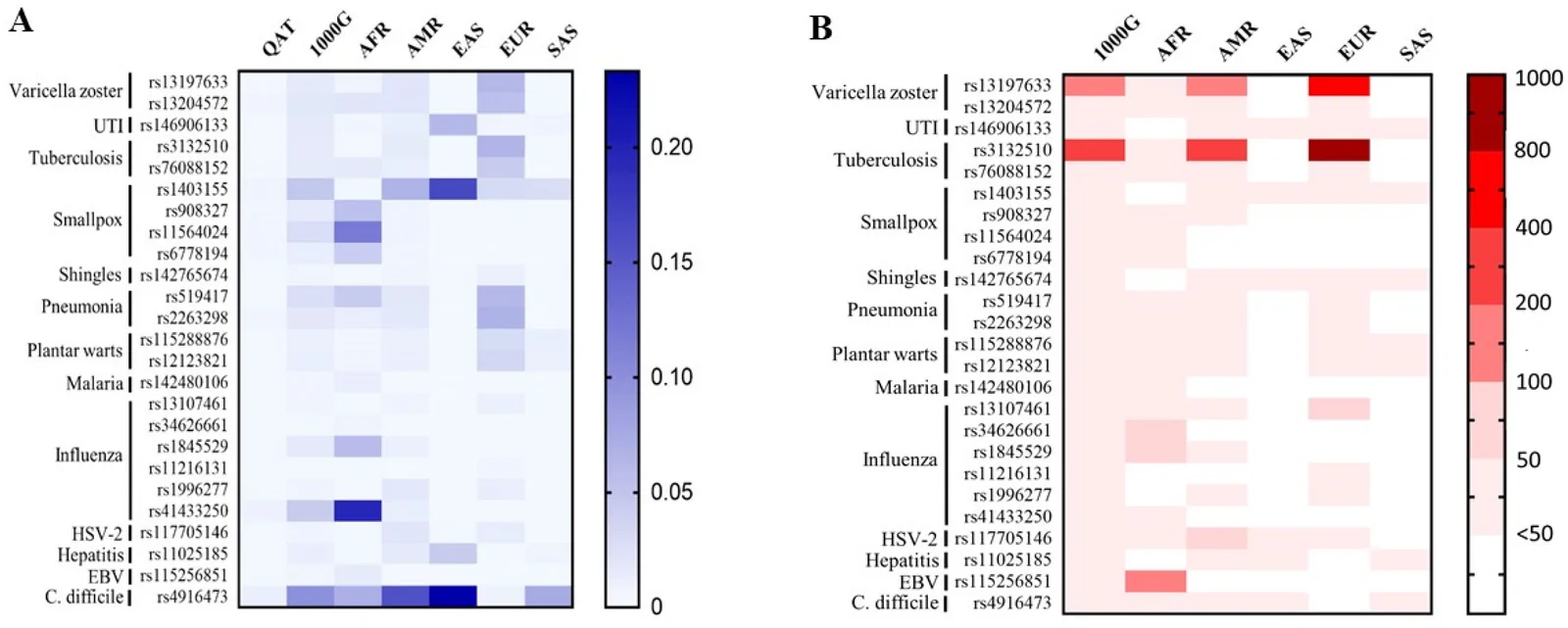
Distribution of infection-related variants in the Qatari population compared to other populations. (**A**). Allelic frequencies of infection-related variants in the Qatari (QAT) population and the 1000 Genome populations. The heatmap shows a comparison in the AFs of the top 25 variants that were markedly different in the Qatari population compared to the 1000 Genome project’s five populations: AFR: African; AMR: American; EAS: Eastern Asian; EUR: European; and SAS: South Asian. UTI, urinary tract infection; HSV-2, herpes simplex virus type 2; EBV, Epstein–Barr virus. Higher color intensity indicates higher allelic frequencies. (**B**). Fold difference in the AF calculated between the Qatari population and each of the 1000G populations for the same set of variants. Higher color intensity indicates larger fold differences.

Initially, we compared the AFs of 883 variants that were associated with infection susceptibility, resistance, severity, progression, clearance, response to treatment, or vaccination. We observed significant variation in the AFs between the Qatari population (*n* = 6047) and other world populations represented by the 1000G project (*n* = 2504). The most significant differences were found in variants related to susceptibility to tuberculosis (TB) and immune response against varicella zoster infections. The frequency of rs3132510 (allele C), which correlates with TB, was 222-fold lower in the QGP population (0.0083%) compared to the global frequency (1.8%). Inter-population comparison indicated other striking differences in the distribution of this variant particularly. In the EUR population, 7.8% carry allele C of this SNP, which is 937 times higher than in Qataris. Similarly, rs13197633-A, which is related to varicella zoster virus glycoproteins (E and I) antibody levels, showed marked AF differences. Carriers of allele A represented 0.017% of the Qatari population, which is 156- and 438-fold lower than the AMR and EUR populations, respectively. Figure 1A shows the AF of the top differentially distributed variants between populations, while Figure 1B demonstrates the fold change (FC) of these variants calculated according to the Qatari population AFs precisely. Importantly, these initial findings provided an avenue for the subsequent detailed analysis, which focused on significantly differing variants (with at least a 2-fold difference in the AF of either allele). These variants were classified based on the associated clinical phenotype. The effect allele, along with the direction of fold change in AF, was then investigated in the QGP cohort and compared to the global frequency.

### 3.2. Genetic Variation in Susceptibility to Infectious Diseases

**Figure 2:**
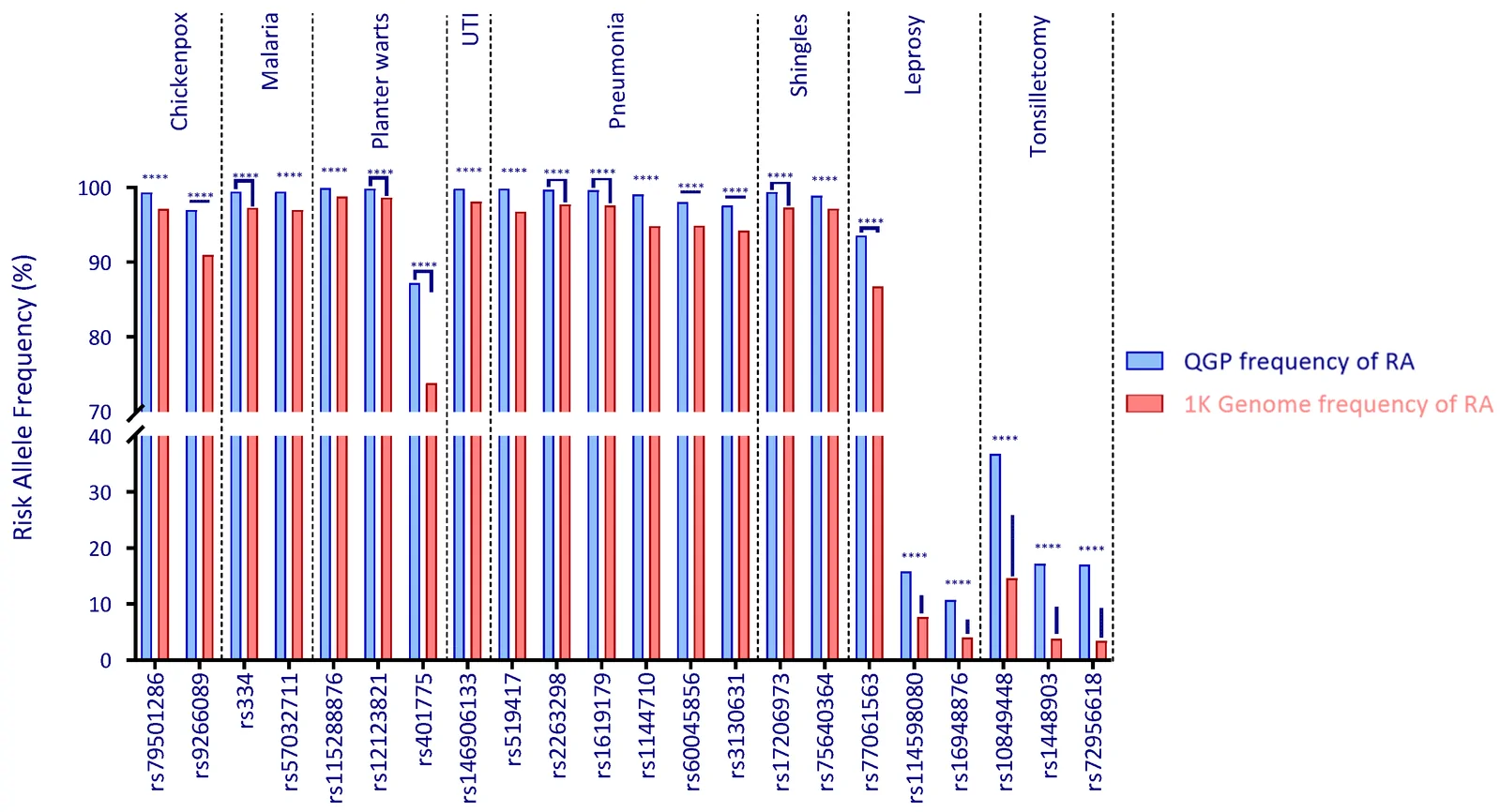
Infection susceptibility variants with increased AF in the Qatari population. The AFs of 22 variants that increase the susceptibility to 8 infections is shown for the Qatari compared to 1000G populations. All these variants exhibited higher AFs among Qataris. The significance of the difference in AF of the risk allele (RA) between the two groups was calculated using the Pearson Chi2 test. To account for multiple comparisons, *p*-values were adjusted using the Benjamini–Hochberg false discovery rate (FDR) method. Statistical significance is indicated as follows: FDR-adjusted *p* < 0.0001 (****). GraphPad Prism 7 was used for analysis and graph plotting.

Out of 883 SNPs included in the analysis, 270 were associated with host susceptibility to a viral, bacterial, or parasitic infection, out of which 52 had considerable AF differences between the Qatari and 1000G populations. Allele frequency analysis based on the reported risk allele, which presumptively increases the susceptibility to infection, indicates that Qataris carry a significantly overrepresented frequency of variants related to multiple infections. This includes chickenpox, malaria, plantar warts, urinary tract infections (UTIs), pneumonia, shingles, leprosy, and tonsillectomy. However, the fold change in the frequency of these risk alleles did not exceed 5-fold. Tonsillectomy-related SNPs (rs72956618-T and rs1448903-G) demonstrated the highest FC in AF (4.97- and 4.5-fold, respectively). The prevalence of rs72956618-T and rs1448903-G in the Qatari cohorts was 17% and 17.2%, compared to 3.4% and 3.8% overall prevalence in the 1000G population, respectively. Allele A of rs10849448, which is also linked to tonsillectomy, was 22.2% higher among Qataris (QGP = 36.9%, 1000G = 14.6%, FC = 2.5). Variants that increase the susceptibility to plantar warts, a condition that is usually caused by human papillomavirus (HPV) infection, were also significantly higher among Qataris (QGP = 0.87, 1000G = 0.74). Additionally, the risk alleles of all SNPs (rs519417, rs2263298, rs1619179, rs1144710, rs60045856, and rs3130631) associated with pneumonia susceptibility were more prevalent among Qataris but with marginal differences in the frequency (2–4%). Similarly, the distribution of other SNPs affecting the susceptibility to leprosy, chickenpox, malaria, and shingles was all enriched in the Qatari genomes, yet with minor fold differences (Figure 2).

On the other hand, the Qatari population carries significantly lower percentages of risk variants associated with TB susceptibility. Three variants (rs3132510-C, rs76088152-A, and rs148844907-A) were significantly less frequent in the QGP cohort, with percentages of 0.008%, 0.132%, and 0.099% compared to 1.84%, 1.82%, and 0.55% in the 1000G, respectively. Another variant that contributed to shingles infection (rs142765674-G) was almost 14-fold less prevalent among Qataris (0.02%) compared to other world populations (0.34%). Moreover, variants linked to hepatitis susceptibility were less common among Qataris. The percentage of rs11025185-A was 0.12% in Qataris compared to 1.5% among others (FC = 12). Another hepatitis-related variant (rs17568725-C) was 19% less frequent in the QGP population (AF = 66.3%), compared with 85.5% in the 1000G dataset.

Although three variants that increase the risk for tonsillectomy showed significantly high frequencies among Qataris, the other eight tonsillectomy-related variants were less frequent (FC = 2–4). Likewise, most malaria-associated variants were less common in the Qatari cohort. While the frequency of rs57032711-G and rs334-T was marginally higher, rs142480106, rs116423146, and rs11036238 risk alleles were remarkably lower in Qataris. The T risk alleles of rs142480106 and rs116423146 were approximately 10- and 5-fold lower in the QGP, respectively, while the G allele of rs11036238 was found in 63.2% of Qataris compared to 85.4% among others.

**Figure 3:**
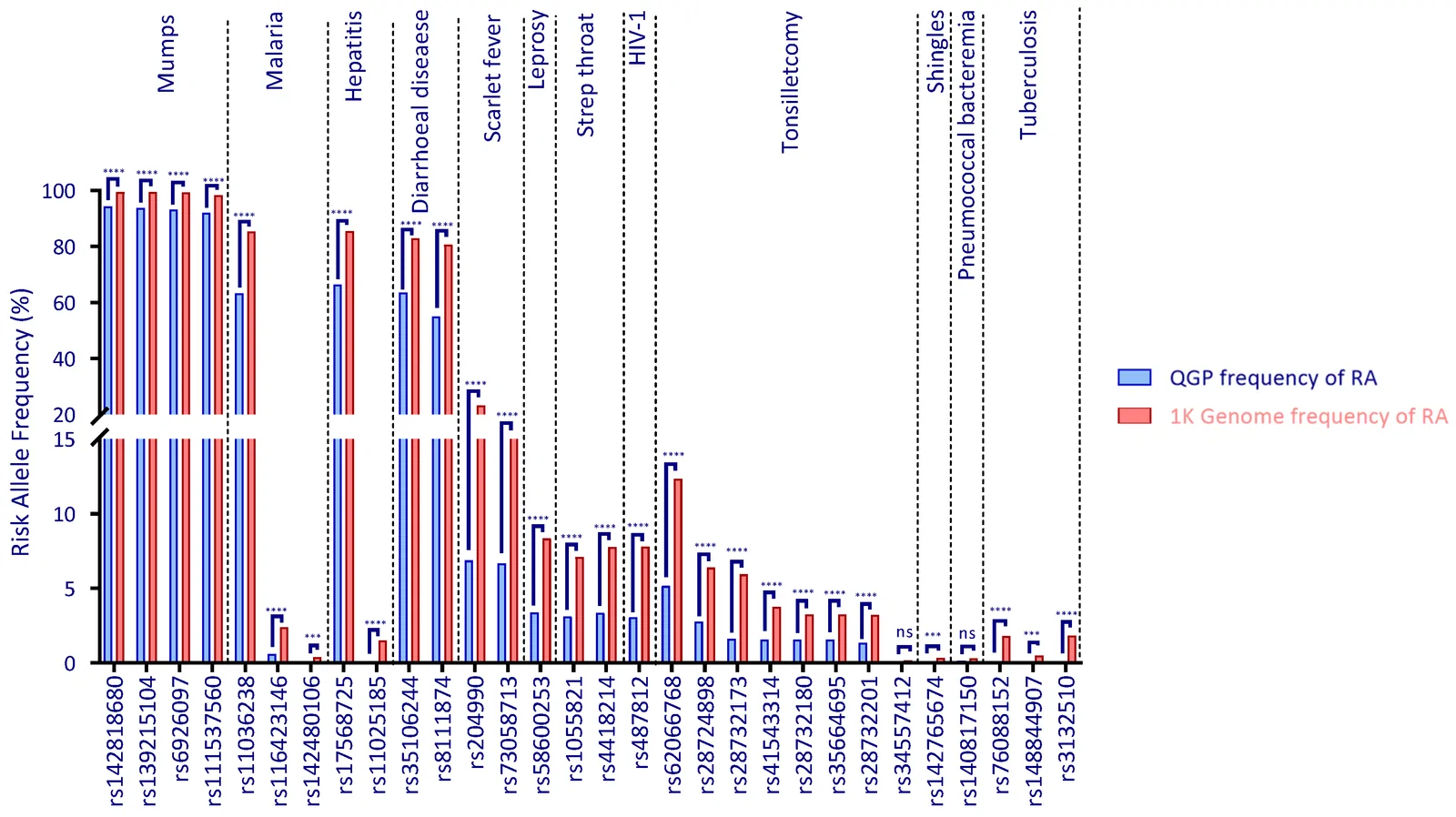
Infection susceptibility variants with decreased AFs in the Qatari population. The AFs of 30 variants that increase the susceptibility to 13 infections are shown for the Qatari population compared to the 1000G populations. All these variants exhibited lower AF among Qataris. The significance of the difference in AF of the risk allele (RA) between the two groups was calculated using Pearson’s Chi2 test. To account for multiple comparisons, *p*-values were adjusted using the Benjamini–Hochberg false discovery rate (FDR) method. Statistical significance is indicated as follows: FDR-adjusted *p* < 0.001 (***), and FDR-adjusted *p* < 0.0001 (****). ns: not significant. GraphPad Prism 7 was used for analysis and graph plotting.

Substantial differences were also observed in variants associated with diarrheal disease, a condition that is caused by several bacterial, viral, and parasitic organisms; most commonly rotavirus and *Escherichia coli* infections [14]. Two diarrheal disease-associated variants, rs8111874-G and rs35106244-C, were less commonly found in the Qatari population, with a 25.5% and 19.4% decrease, respectively. Additionally, a lower percentage of rs204990-A and rs1055821-T was observed among Qataris. These variants increase the susceptibility to scarlet fever and strep throat, which are both caused by group A streptococcus infections. Qatari genomes also exhibited a lower enrichment in the AFs of SNPs associated with mumps, leprosy, and HIV but with equal or less than 3-fold change (Figure 3).

### 3.3. Genetic Variation in Infection Severity or Chronicity

**Figure 4:**
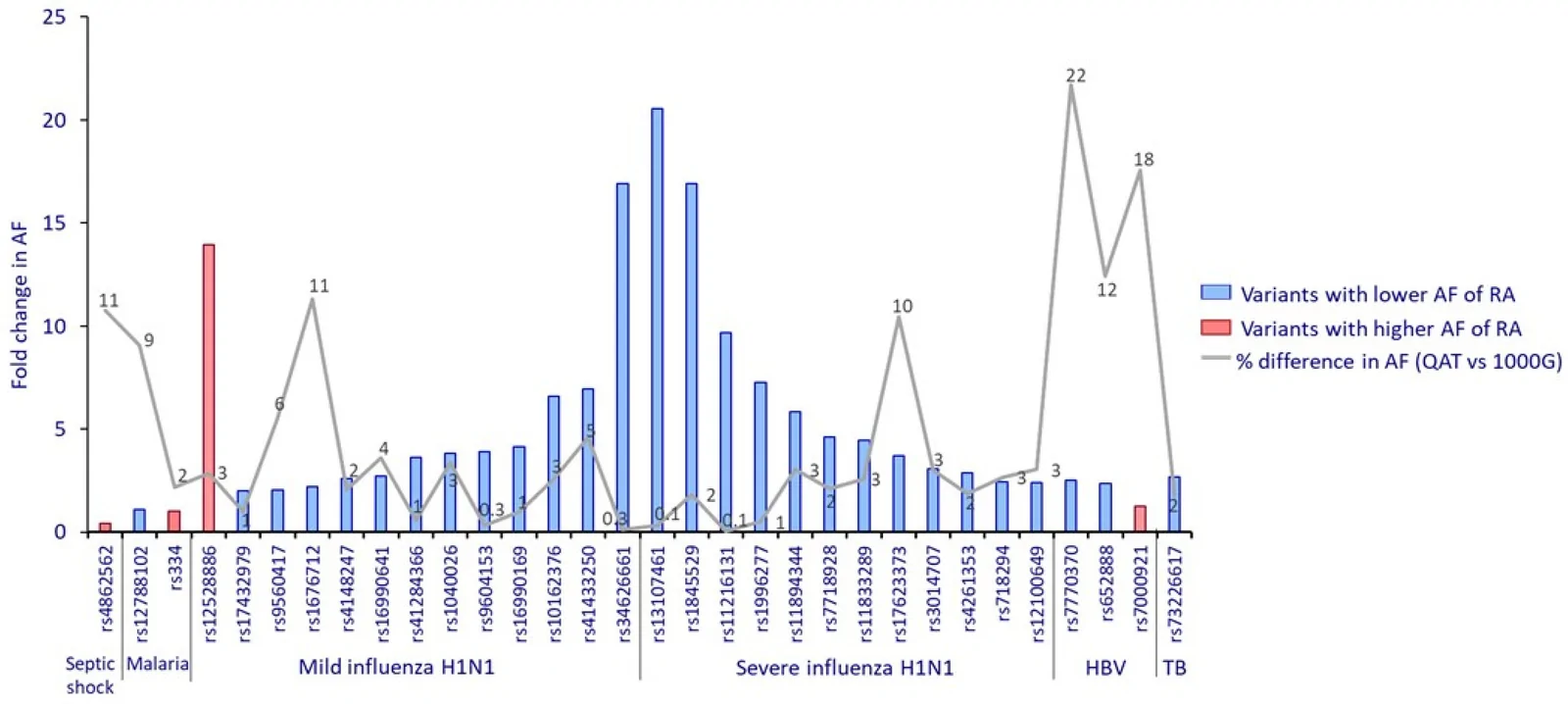
The fold difference in the AFs of variants related to infection severity in the Qatari compared to the 1000G populations. Fold differences in the AFs calculated between the Qatari and the 1000G populations for the top 32 variants associated with infection severity or chronicity of 6 infections are shown. Bars colored in red indicate a higher AF of the risk allele among Qataris, while blue colored bars indicate a lower AF of the risk allele among Qataris. AF, allele frequency; RA, risk allele; HBV, hepatitis B virus; TB, tuberculosis.

We further analyzed host variants that influence the progression to severe or chronic infection. A total of 92 variants associated with nine infections were analyzed. While the AFs of variants contributing to septic shock, malaria, influenza, HBV, and TB severity/chronicity had ≥2 FC, pneumococcal meningitis-, HIV-, HCV-, and SARS-CoV-2-related variants showed a comparable population genetic distribution. Remarkably, most severity/chronicity-related variants were considerably less common in the Qatari population. For instance, rs13107461, which was linked to severe influenza H1N1 infection, showed a 20.5-fold lower prevalence among Qataris compared to the 1000G cohort. In addition, except for rs12528886, all other H1N1 severity-related variants (*n* = 23) exhibited lower percentages in the Qatari population. On the contrary, the distribution of variants related to mortality from septic shock and HBV persistence was higher among the Qatari population (18% and 84% compared to 7.9% and 66.5%, respectively) (Figure 4).

### 3.4. Genetic Variation in Antibody Response to Infections

Amongst GWAS catalog associations on infectious diseases, numerous had correlated host genetic polymorphisms with antibodies response following infections. Covering 11 bacterial and viral pathogens, 41 SNPs were selected for the analysis based on the associated statistical significance. The most significant differences between the QGP and the 1000G datasets were found in variants associated with Epstein–Barr virus (EBV). The frequencies of the minor (effect) alleles of rs115256851 and rs114676416 were 27.8- and 2.7-fold lower in the Qatari compared with the 1000G cohorts, respectively. Two other variants (rs4248166 and rs2854275), whose major alleles contribute to EBV antibody response, were also differentially distributed. A total of 93.8% of Qatari genomes harbor allele T of rs4248166, compared to 82.2% in other populations. On the other hand, the percentage of rs2854275-C was 8% lower in Qataris (AF = 85.5%) compared to others (93.5%). In contrast to EBV, the distribution of variants associated with other herpes viruses, including herpes simplex virus type 2 (HSV-2) and human herpesvirus 7 (HHV-7), was comparable between different populations.

**Figure 5:**
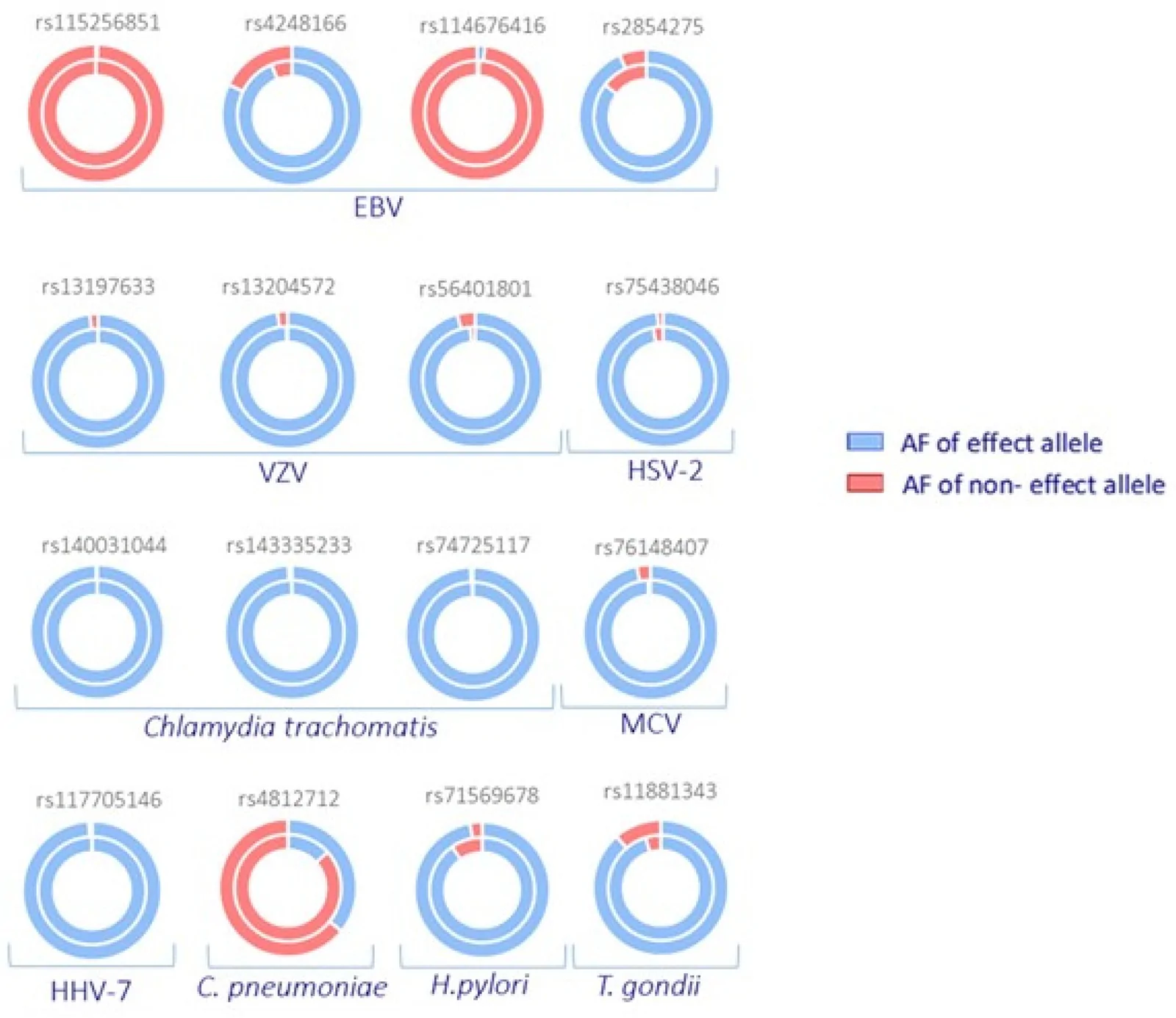
Allelic frequencies of antibody response-related variants in the Qatari compared to the 1000G populations. The AFs of the top 16 variants that are associated with the antibody response against 9 viral and bacterial infections are shown. Each circle represents either the Qatari (outer circle) or the 1000G population (inner circle). The color coding corresponds to the allele type (effect alleles: red; non-effect alleles: blue). EBV: Epstein–Barr virus; VZV: varicella zoster virus; HSV-2: herpes simplex virus-2; MCV: molluscum contagiosum virus; HHV-7: human herpesvirus 7; *C. pneumoniae*: *Chlamydia pneumoniae*; *H. pylori*: *Helicobacter pylori*; *T. gondii*: *Toxoplasma gondii*.

Host variants related to the antibody response to bacterial infections demonstrated variable population differences. The most significant difference in allelic percentages was observed in *Chlamydia pneumoniae* (rs4812712, 22.5%), followed by *Helicobacter pylori* (rs71569678, 6.8%). On the other hand, *Chlamydia trachomatis*-related SNPs (rs140031044, rs143335233, and rs74725117) varied insignificantly in their allelic frequencies. Figure 5 illustrates the differences in the AFs of the top variants associated with antibody response.

### 3.5. Genetic Variation in Drug Response to Infections

**Figure 6:**
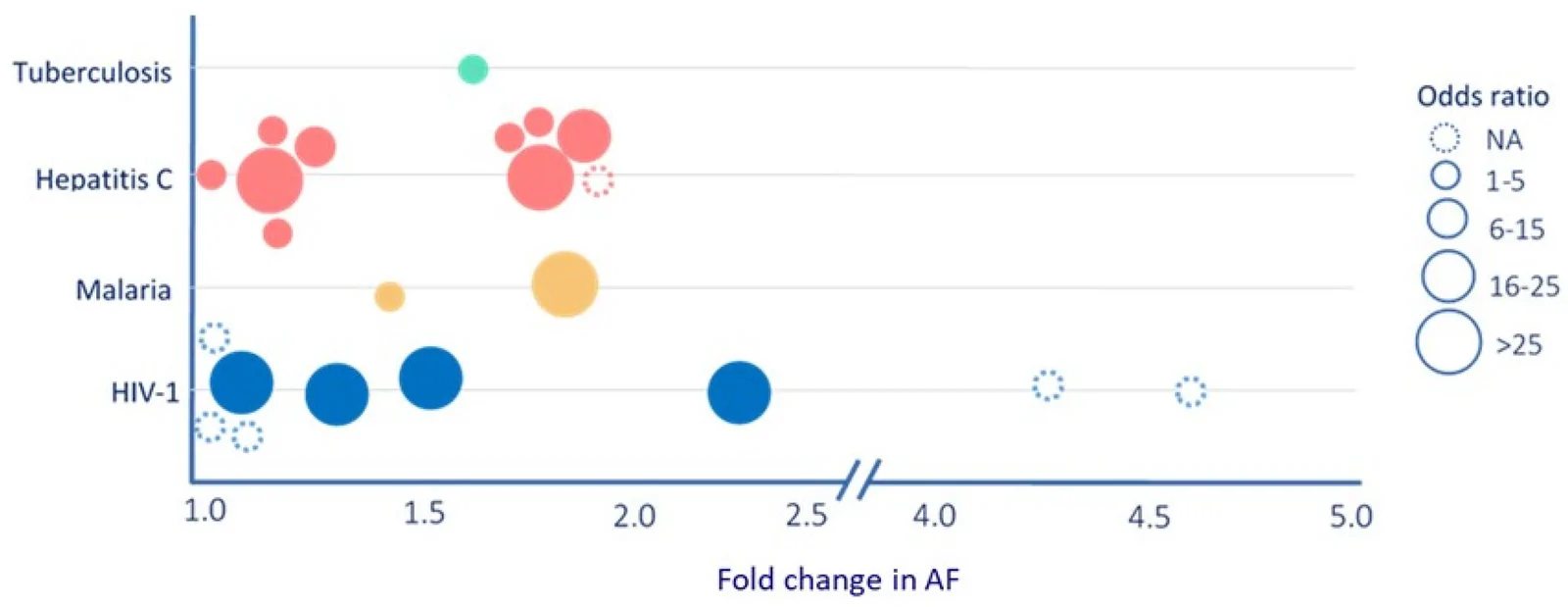
Fold differences in the AFs of drug response-related variants in the Qatari compared to the 1000G populations. The AFs of the top 22 variants that are associated with response to treatment against four infections (TB, HCV, malaria, and HIV-1) are shown. Each circle represents one SNP. NA: variants with no information on the odds ratios.

Despite their large effect size compared to other phenotypes, GWASs in the treatment of IDs were very scarce. Here, we investigated whether there is a population-distinct distribution of variants that are involved in the treatment of TB, HCV, malaria, and HIV-1. Overall, we found no significant differences in the allelic frequencies of these variants between the Qatari and the 1000G population. Only three SNPs related to HIV-1 susceptibility to d4T/ddI-associated peripheral neuropathy, rs9501753, rs7568498, and rs266095, showed FCs of 4.6, 4.3, and 2.4, respectively. Nonetheless, although most drug response-related variants showed comparable allelic frequencies between the Qatari and the general world population, large differences were observed in comparison to Africans and East Asians, specifically. Most importantly, multiple variants had a high odds ratio (up to 56 odds), as observed with malaria (Tafenoquine efficacy), HCV (thrombocytopenia in pegylated interferon and ribavirin therapy), and HIV-1 (ddI/d4T therapy associated neuropathy) (Figure 6).

### 3.6. Genetic Variation in Response to Vaccination Against Infectious Diseases

**Figure 7:**
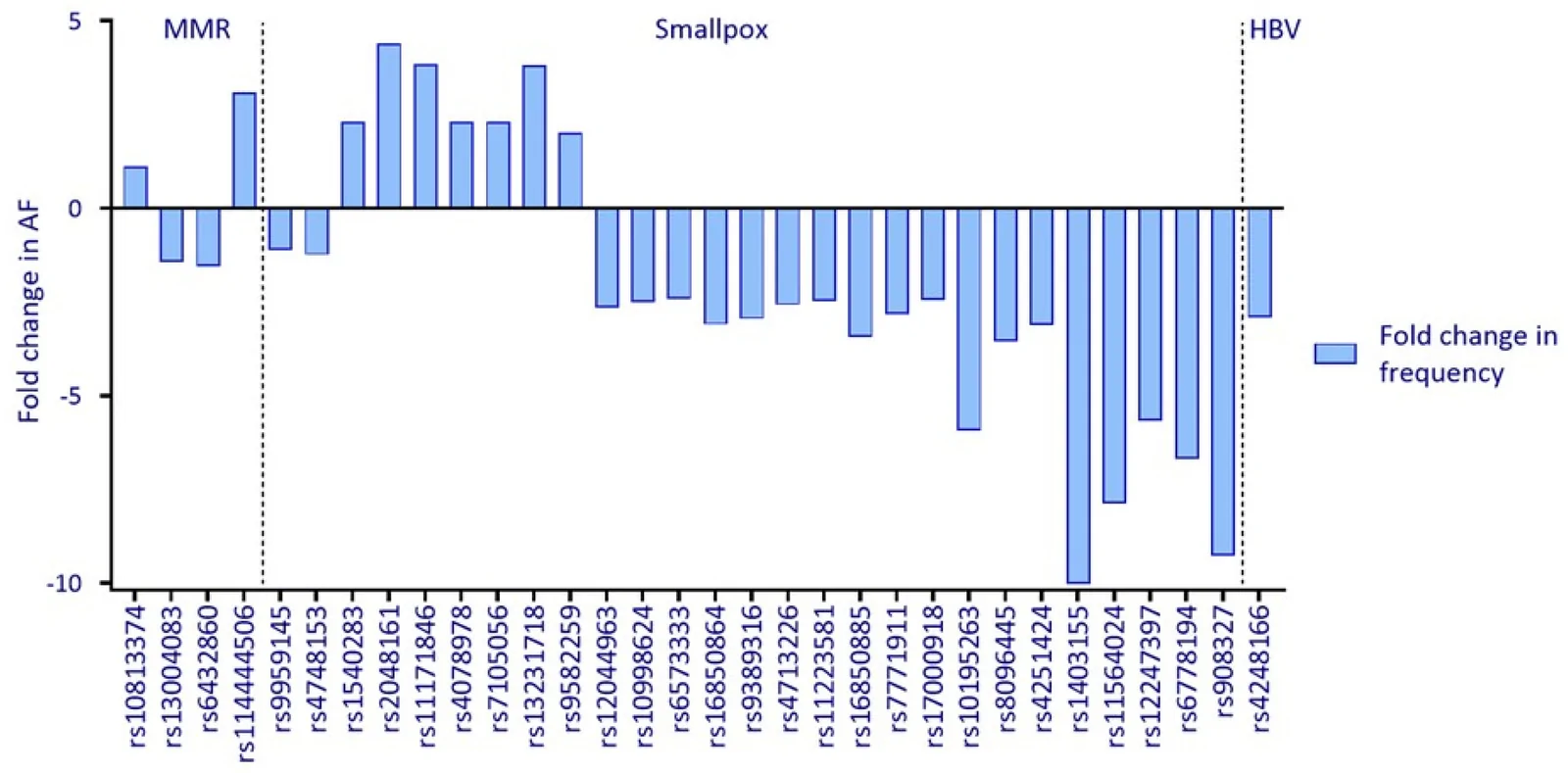
The fold difference in the AFs of variants related to vaccine response in the Qatari compared to the 1000G populations. Fold differences in the AFs calculated between the Qatari and the 1000G populations for 32 variants associated with response to MMR, smallpox, and HBV vaccinations are shown.

We found 10 genome-wide studies on the immune response or adverse events following vaccination in the GWAS catalog. A total of 80 host variants were significantly associated with vaccine response, of which 32 exhibited a variable inter-population distribution (FC ≥ 2) (Figure 7). These variants were explicitly related to MMR, smallpox, and HBV vaccines. The most substantial differences in the AF were found in variants related to the smallpox vaccine, reaching up to a 10-fold change. Most of these variants are associated with cytokine response following smallpox vaccination and demonstrated a lower frequency of the effect allele. In addition, one variant (rs4248166-C), which is associated with HBV vaccine response, was around 3-fold less common in the Qatari population. The majority of MMR vaccine-related SNPs, on the other hand, were distributed with less variability. However, rs114444506 (risk allele C) was significantly higher among Qataris (3.2%) compared to the 1000G population (1%). This variant is associated with an increased risk of MMR vaccine-related febrile seizures.

### 3.7. Genetic Variation in Other Phenotypes Related to Infectious Diseases

Very few studies reported host variants associated with virus clearance or resistance against infections with GWAS significance (7 studies, 13 SNPs). The QGP and 1000G cohorts demonstrated comparable frequencies for most variants (FC < 2). Only the AF of rs186873296, which is related to resistance to severe malaria, varied by around 9-fold between the two populations. In addition, rs3077, which is associated with HBV clearance, showed an 18.2% difference in the allelic distribution (Table 1).

Similarly, we did not observe large-fold differences in the minor alleles of 21 SNPs that are associated with virus-induced progression to cancer. Yet, the difference in the percentage between the Qatari and 1000G populations reached 35% (Table 2).

Only two viruses, HIV and HCV, were studied in the context of the host genomics of viral load. We analyzed 84 SNPs significantly associated with pre-treatment viral load in HIV (*n* = 83) or HCV (*n* = 1). Out of 84 variants, 35 (all related to HIV) demonstrated considerable variability in the AFs between the QGP and 1000G cohorts. The FC ranged between 2 and 6.1, while the AF percentage differed by 2.1 to 21.5% (Table 3).

## 4. Discussion

In this study, we provided a comprehensive screening of common infection-related host variants in a cohort of the native Qatari population. The availability of a large set of whole-genome sequences from the QGP’s first phase (*n* = 6047) enabled us to study the frequency of host genetic variants contributing to the response to infectious disease in a Middle Eastern Arab population. Razali et al. showed previously that the QGP dataset broadly overlaps with samples from other regions of Arabia, the Levant, Iraq, Iran, Turkey, and North and East Africa, indicating the genetic diversity of the QGP cohort and, therefore, its suitability to represent the wider Middle Eastern region [33]. Overall, our analysis revealed distinct characteristics of the Qatari population in the distribution of variants related to IDs. This pattern may reflect the unique genetic architecture of the population, including the influence of high consanguinity and founder effects [34].

The Qatari population is genetically diverse and comprises multiple ancestral lineages that reflect the complex demographic history of the Arabian Peninsula. The region has historically served as a crossroads of human populations, and its genetic landscape has been shaped by population divergence, founder effects, admixture, and long-standing population structure. These demographic and population genetic factors may contribute to the observed distribution of infectious disease-associated variants and should be considered when interpreting population-level differences in allele frequencies.

Compared to various world continental regions (1000G dataset), we observed a notable allele similarity between the QGP and South Asian (SAS) populations, specifically in variants related to many IDs. In accordance with that, Razali et al.’s assessment of the QGP’s population structure reported that the highest allele sharing was with Africans, followed by the SAS population [33]. Indeed, their analysis included a considerably more extensive set of variants, which enabled an in-depth comparative analysis of genetic structure and ancestry against diverse world populations.

Comparing the distribution of variants that contribute to infection susceptibility revealed a considerably lower frequency of most susceptibility risk variants in the QGP cohort, particularly variants related to TB, hepatitis, and diarrheal disease. The decrease in allelic frequency reached 222-fold, as observed with a TB-related variant, rs3132510. In fact, the marked decrease in TB susceptibility variant frequency is consistent with, though not confirmatory of, the low circulation of TB among Qatari nationals. Although the incidence of TB is the highest in the Gulf region, this is mainly attributed to the continuously increasing number of migrant laborers [35]. A previous report indicated that while the incidence rate of TB is around 42/100,000 in Qatar, 90% of the patients are non-Qatari male workers coming from high TB-endemic areas [36]. Consistent with this epidemiological pattern, most variants nominally associated with malaria susceptibility were less common. Malaria had an incidence rate of 17.3/100,000, none of which was from Qatari origin. Mostly, malaria cases in Qatar are imported from malaria-endemic countries such as India, Nepal, Pakistan, and Sudan [37,38]. In line with that, most variants increasing the susceptibility to malaria were remarkably less common in the Qatari cohort, with up to a 9-fold difference in the allelic frequency. In addition to TB and malaria, we found that host genetic variants related to viral hepatitis exhibited significantly reduced prevalence in the QGP dataset (rs11025185-A and rs17568725-C), reaching a 12-fold decrease. Qatar is considered one of the countries with low HCV rates in the Middle East and North Africa (MENA) region, with a prevalence of 2% in the general population (2015) [39]. A report by the MoPH indicated a 25.3/100,000 incidence rate for HCV in 2018 [37]. On the other hand, the annual incidence of HBV in Qatar is 19.8 per 100,000 individuals, where Qatari nationals represent only 6% of the cases [40]. This incidence rate increased to 28.9/100,000 in 2018, yet it is still considered relatively low compared to other MENA countries such as Egypt and Yemen [37,41].

Although hepatitis viruses were extensively studied in the context of host genetic variability, these studies were primarily clustered in populations other than MENA-region populations, except for very few studies conducted in Saudi Arabia and Egypt [42,43,44]. Importantly, populations of the MENA region are the most affected by hepatitis globally [45]. Moreover, the variability in hepatitis prevalence rates in this region particularly could reflect a combination of host genetic, environmental, demographic, healthcare, and socioeconomic factors. We have previously conducted a GWAS on the hepatitis E virus (HEV) in Qatar, which circulates at a higher rate (12.3%) compared to HBV and HCV and can progress to chronicity and lead to multi-organ complications. We found several variants associated with HEV seroprevalence and antibody levels in the Qatari population, many of which mapped to genes involved in immune regulation [46]. Given the differences in haplotype structure and AFs among different ancestries, implementing GWASs in the MENA populations is essential to further improve our understanding of hepatitis virus–host interactions.

A substantial reduction in the frequency of variants related to diarrheal disease among Qataris was also observed (rs8111874 and rs35106244 with 25.5% and 19.4% decrease, respectively). Notably, rs8111874-G and rs35106244-C exhibit a high ancestry-specific distribution, ranging from 0.57 and 0.58 in Europeans to 0.993 and 0.996 in East Asians, respectively. These variants are linked to the *FUT2* gene, which has been associated with susceptibility to different gastrointestinal infections such as norovirus and rotavirus [47,48]. Interestingly, *FUT2* harbors population-specific inactivating variants, such as the stop mutation W154X (rs601338) in Caucasians (Europeans and Iranians) and Africans and the missense variant A385T (rs1047781) in Asians [49]. It has been reported that *FUT2* has a complex pattern of natural selection, evidenced by variants with a long history of balancing selection among Eurasian and African populations and the positive selection of one fast-spreading variant in East Asia [49]. This may partially explain the significant differences observed in the allelic frequency of *FUT2* SNPs in the Qatari population compared to the global frequencies. Although it can be caused by a wide range of viral, bacterial, and parasitic agents, rotavirus and *Escherichia coli* are the most common etiological agents of diarrheal disease [14]. The crude incidence rate of rotavirus in Qatar is estimated at 8.1/100,000 per year [37]. We have shown in a previous study on acute gastroenteritis (AGE) in pediatrics that rotavirus was responsible for 28.4% of total AGE cases (*n* = 812) [50]. Interestingly, the prevalence of rotavirus infection was higher among Qatari patients (36.3%) in comparison to other MENA nationalities, such as Egyptians (11.2%). On the other hand, estimates on *E. coli* infections among the Qatari population are not available. Collectively, it is unfeasible to conclude whether the low frequency of diarrheal disease-related host variants in the Qatari population is actually translated into disease incidence, considering the contribution of multiple pathogens in diarrheal disease and the lack of sufficient nationality-adjusted epidemiological reports.

We also found a 13-fold decrease in rs142765674-G, a variant previously linked to shingles susceptibility. This variant has a population prevalence of 0.02% in Qataris compared to 0.34% globally. The substantially lower frequency of this susceptibility variant may reflect population-specific differences in the genetic architecture associated with herpes zoster (HZ) susceptibility. Supporting a potential role for host genetics, HZ has previously been reported to be approximately 50% more common among individuals of non-Hispanic White ancestry than among those of African American ancestry [51], indicating that ancestry-related genetic factors may contribute to differences in disease risk.

Population-level HZ incidence is also influenced by non-genetic factors. After accounting for population representation, the mean incidence of HZ was estimated to be 79/100,000 among Qataris and 101/100,000 among expatriates in Qatar [52]. One contributing factor may be Qatar’s early implementation of the varicella vaccine as part of its national immunization program in 2002 [53]. Thus, most of the children in the country are immunized against varicella zoster virus (VZV), and this could have contributed to the lower incidence rates observed in the country.

Furthermore, a consistent pattern of lower frequency of group A streptococcus susceptibility variants (*n* = 9), specifically those associated with scarlet fever, tonsillectomy, and strep throat, was observed in the QGP cohort (up to 16% decrease in AF). Although we found three tonsillectomy-related variants significantly increased in the Qatari population, seven other variants had significantly lower frequencies. Together, this could indicate a lower susceptibility to a wide range of diseases caused by group A streptococcus among Qataris, although this requires additional investigation.

Contrarily, we observed a higher prevalence of multiple risk variants that increase the susceptibility to chickenpox (*n* = 2), leprosy (*n* = 3), plantar warts (*n* = 3), UTIs (*n* = 1), and pneumonia (*n* = 6) in the Qatari cohort. This increase, however, was at most 5-fold. Additionally, although most tonsillectomy variants (*n* = 8) exhibited lower frequencies among Qataris, three variants were more prevalent, including rs10849448. This variant was 2.5-fold higher among the Qatari cohort, with a frequency of 36.9% compared to 14.6% in the 1000G data. Other than its role in tonsillectomy, homozygosity for allele A of rs10849448 has been reported previously to increase the risk of non-small-cell lung carcinoma (NSCLC) by 1.8 odds [54]. Therefore, it might be of great benefit to interpret this variant—along with others—in a clinical context by linking the phenotypic data and participants’ medical records to their genotypes.

In concordance with the decrease in susceptibility variants, we also found noticeably low frequencies of most severity-related variants (28 out of 32). This pattern may be consistent with a lower genetic predisposition to the analyzed infections, though this cannot be inferred directly from allele frequency alone. In addition, this highlights the need for regional GWASs that could reveal novel population-specific risk loci. Moreover, accurate and recent figures on the prevalence of many infectious diseases among Qatari nationals still need to be included. The available data was, therefore, insufficient to provide a clear epidemiological picture of all infections in correlation with population genetics.

Our analysis of variants associated with antibody response to bacterial and viral infections revealed distinct variations related to EBV, *Chlamydia trachomatis*, and *Helicobacter pylori*. Interestingly, the two significantly different anti-EBV-related SNPs (rs115256851 and rs114676416) were reported as African-specific variants [55]. rs115256851 is monomorphic in all populations except Africans (AF = 1.74%), while rs114676416 is monomorphic in all populations except Africans (6.81%) and Americans (0.4%). Our data indicated the presence of these two variants in 0.017% and 0.67% of Qatari individuals, respectively. This again points out how the inclusion of previously unrepresented ancestries provides a broader insight into population genetics structure. Furthermore, although multiple studies emphasized the central and pathogen-specific role of human gene variation (including the human leukocyte antigen, HLA) in the modulation of humoral immune response to viral antigens, the limited number of studied pathogens, the unknown impact of pathogen genetic diversity, and infection time/recurrence all contribute to the immense knowledge gaps that still exist [56,57,58].

One of the main challenges of GWASs is that complex phenotypes are generally modulated by a large number of genetic variants, each of which contributes to a relatively small effect. In fact, odds ratios for a typical GWAS range between 1.1 and 1.2 [59]. Nonetheless, the case is different with pharmacogenomics-related variants, which have much more prominent effect sizes [60]. In agreement with that, we analyzed 22 variants associated with drug response to TB, HCV, malaria, and HIV-1 infections, out of which 15 variants had substantially high odds ratios (2–56 odds). The most considerable differences in AF between QGP and other populations were observed in three SNPs related to antiretroviral peripheral neuropathy, a common complication during HIV treatment. While two of them lacked information on odds ratios, one variant (rs266095) with extremely high odds (OR = 56) was two-fold higher among the Qatari population. Qatar remains an HIV low-prevalence country, with an average incidence rate of 0.69/100,000 (2000–2016) [61]. However, having a distinct distribution of variants associated with complications of antiretroviral therapy with high odds requires further investigation in Qatari patients. These variants may represent candidates for future investigation as predictors of drug toxicity risk, pending validation in phenotype-linked cohorts. Eventually, this will facilitate the development of a tailored therapy based on the patient’s genotype, leading to a more effective antiviral course.

Population-based studies brought attention to the comparatively high rates of vaccine failure and the potential contribution of genetic factors. It is found that 1–10% and 5–10% of people who receive the measles or HBV vaccine do not develop protective immunity, respectively [62,63]. Additionally, 2–5% of people who receive the rubella vaccine do not seroconvert. Even among those who respond to vaccination, there is a high degree of immunological variability, which is suggested to be heritable [64]. Considering this, we analyzed 80 SNPs that were significantly linked to vaccine response. Variants related to smallpox, HBV, and MMR vaccine response exhibited considerable population differences in the QGP cohort that reached 10-fold. Importantly, these SNPs were not only associated with the immune response following vaccination but also with vaccine-adverse events. For instance, rs114444506, which doubles the risk of febrile seizures after MMR vaccination (OR = 2.09), is present in 3.2% of Qataris compared to 1% among the 1000G cohort [65]. Since febrile seizures represent a serious adverse event following the MMR vaccine, it is essential to understand their genetic basis in diverse ancestry populations.

Genetic variants associated with other phenotypes related to infectious diseases, such as viral clearance, viral load, and virus-induced progression to cancer, also showed a variable distribution in the Qatari population. Nonetheless, these findings will need to be interpreted in a clinical context to gain a better insight into their impact on disease outcomes.

Inter-population differences in the allelic frequency are a consequence of several processes, including ecological selection pressures that probably determined many occasions of local adaptation [66]. Since the migration of humans out of Africa, they have been exposed to diverse climates, food sources, infectious agents, and other environmental challenges [66]. Pathogens have mainly been considered as one of the most potent forces that apply a remarkably selective pressure on human populations, contributing to the existing diversity of many genes [67,68]. For instance, a very recent report provided a mechanistic basis supporting the hypothesis that Gaucher disease protects against TB among Ashkenazi Jews [69]. Interestingly, Gaucher disease, due to a mutation *GBA1* N370S that increases resistance to TB, is widespread in Ashkenazi Jews. This provides plausibility for TB-driven selection over the centuries. It also highlights the value of exploring the interaction between population genetic diversity and the history of infectious diseases in the region to understand the factors that drive selection and shape susceptibility or resistance.

Several key limitations of this study warrant consideration. First, the QGP cohort is substantially larger than the individual populations represented in the 1000 Genomes Project, which may influence statistical power and the interpretation of significance testing. In addition, differences in ancestry composition, demographic history, founder effects, population structure, and the relatively high levels of consanguinity reported in Arabian populations may contribute to the observed allele frequency differences. Second, the QGP cohort included in this study is restricted to adults (≥18 years), precluding the assessment of genetic factors that may influence susceptibility to infectious diseases during childhood. Third, because most analyzed SNPs were originally identified through GWASs conducted in predominantly European-ancestry populations, differences in haplotype structure and linkage disequilibrium patterns may affect the tagging efficiency and effect sizes of these variants in the Qatari population. Moreover, the variant calls were generated using the QGP first-phase analytical pipeline (GRCh37/GATK v3.3), although the use of newer reference assemblies and variant-calling pipelines may result in minor differences. Most importantly, the absence of linked phenotypic and clinical outcome data prevents us from directly validating whether the observed allele frequency differences translate into measurable differences in disease incidence, severity, or treatment response among the Qatari population. The linkage of QGP genotype data with Qatar Biobank clinical records and national disease registries remains the critical next step.

## 5. Conclusions

In this study, we provided a description of the genetic architecture of an ancestrally diverse and large Arabian cohort with respect to IDs. We report a unique distribution of infection-related host variants among the Qatari population. However, these findings should not be interpreted as evidence of differences in disease susceptibility or clinical outcomes without functional validation and population-specific association studies. Moving forward, it is critical to validate these findings primarily using phenotypic clinical patients’ data. Furthermore, the linkage of QGP genotype data with clinical phenotypic records could, in the future, support the development of polygenic risk scores (PRS) to predict individual susceptibility to infectious diseases, representing a potential step toward precision public health and personalized risk assessment. Additionally, we propose utilizing the extensive genome sequencing data of the QGP to perform GWASs on infections relevant to the Qatari population’s health burden to detect unique loci contributing to ID spread in the region. Future work could prioritize these variants by prevalence and effect size to explore their potential utility in identifying high-risk individuals, pending validation through linked clinical and phenotypic data. The identification of population-prevalent infectious disease-associated variants may also inform future risk stratification strategies, targeted prevention programs, and vaccine prioritization approaches within the Qatari population.

## Figures and Tables

**Table 1:** Common single-nucleotide polymorphisms associated with host resistance or viral clearance.

Phenotype	CHR	POS	SNP ID	REF	ALT	ALT_AF (Qatari)	ALT_AF (1000G)	OR/BETA	Change in AF (%)	Ref
Hepatitis C viral clearance	19	39248515	rs74597329	T	G	0.32	0.37	3.28	−5.25	[15]
	6	32692805	rs2647006	C	A	0.32	0.28	1.79	4.18	
	6	32703120	rs2647051	C	T	0.32	0.27	1.79	4.58	
	6	32693516	rs2858320	G	A	0.34	0.34	1.96	0.58	[16]
	19	39248515	rs74597329	T	G	0.32	0.37	2.19	−5.25	
	6	33065245	rs3077	A	G	0.26	0.44	1.82	−18.21	[17]
	6	33087084	rs9277535	A	G	0.28	0.33	1.64	−4.94	
Resistance to severe malaria	4	143781321	rs186873296	A	G	0.01	0.00	1.49	1.18	[18]
	9	133257521	rs8176719	T	C	0.30	0.34	1.48	−3.94	[19]
	1	203684896	rs10900585	T	G	0.13	0.17	1.64	−4.25	
	11	5496926	rs372091	G	A	0.00	0.02	2.17	−1.57	
Resistance to tuberculosis	10	126659538	rs17155120	C	T	0.13	0.16	0.5	−3.15	[20]
	5	159263943	rs4921437	C	T	0.21	0.14	0.374	7.02	[21]

CHR: chromosome number; POS: genomic position; REF: reference allele; ALT: alternative allele; ALT_AF: alternative allele frequency; OR: odds ratios; BETA, beta coefficient.

**Table 2:** Common single-nucleotide polymorphisms associated with virus-induced progression to cancer.

Phenotype	CHR	POS	SNP ID	REF	ALT	ALT_AF (Qatari)	ALT_AF (1000G)	OR/BETA	Change in AF (%)	Ref
Epstein–Barr virus-associated Hodgkin’s lymphoma	6	31479019	rs2248462	G	A	0.15	0.46	1.64	−31.20	[22]
	6	32465390	rs2395185	G	T	0.38	0.11	1.82	26.76	[22]
	5	132660272	rs20541	G	A	0.17	0.22	1.47	−4.58	[22]
Hepatitis B-associated hepatocellular carcinoma	7	90689160	rs10272859	G	C	0.29	0.20	1.28	8.28	[23]
	6	29971803	rs2523961	G	A	0.14	0.29	1.91	−15.45	[24]
	1	10325413	rs17401966	A	G	0.19	0.18	1.64	1.30	[25]
	6	32632222	rs9272105	G	A	0.44	0.23	1.28	20.45	[26]
	6	32698518	rs9275319	A	G	0.17	0.52	1.49	−34.99	[27]
	21	29773850	rs455804	C	A	0.21	0.20	1.19	0.73	[26]
	6	30103160	rs1110446	C	T	0.20	0.20	1.93	0.79	[24]
	2	191099907	rs7574865	G	T	0.25	0.29	1.21	−4.24	[27]
Hepatitis C-induced hepatocellular carcinoma	6	31398818	rs2596542	C	T	0.44	0.16	1.39	27.93	[28]
	6	32711222	rs9275572	G	A	0.34	0.23	1.3	10.63	[28]
Hepatitis C-induced liver cirrhosis	6	32411959	rs9405098	G	A	0.07	0.27	1.37	−19.86	[29]
	6	32421871	rs3135363	A	G	0.15	0.15	1.37	−0.25	[29]
	6	32433302	rs3129860	G	A	0.07	0.29	1.3	−22.28	[29]
	6	32400310	rs3817963	T	C	0.25	0.42	1.3	−17.17	[29]
	6	32347950	rs910049	C	T	0.21	0.26	1.46	−4.19	[29]
Hepatocellular carcinoma in post-HCV eradication	4	166008836	rs17047200	A	T	0.14	0.23	2.37	−8.21	[30]
Human papillomavirus-associated cervical cancer	5	91087644	rs59661306	A	G	0.17	0.26	1.3	−8.20	[31]
	7	54380269	rs7457728	G	C	0.25	0.34	1.19	−8.27	[31]

CHR: chromosome number; POS: genomic position; REF: reference allele; ALT: alternative allele; ALT_AF: alternative allele frequency; OR: odds ratios; BETA, beta coefficient.

**Table 3:** Common single-nucleotide polymorphisms associated with viral load.

Phenotype	CHR	POS	SNP ID	REF	ALT	ALT_AF (Qatari)	ALT_AF (1000G)	OR/BETA	Change in AF (%)	Effect on Viral Load	FC	Ref
Pre-treatment viral load in HIV-1 infection	1	19308470	rs859208	C	G	0.02	0.10	0.42	−7.79	Increase	4.34	[32]
	1	32362000	rs57541735	G	C	0.02	0.05	0.44	−3.66	Increase	3.12	
	1	34749623	rs626096	C	T	0.02	0.06	0.41	−3.49	Increase	2.42	
	1	164652278	rs2800778	G	T	0.03	0.09	0.53	−6.52	Decrease	3.37	
	1	242242264	rs429360	C	T	0.02	0.06	0.48	−4.75	Decrease	3.96	
	1	244750932	rs113974007	T	C	0.03	0.08	0.35	−4.60	Increase	2.54	
	2	28788221	rs10182825	G	A	0.02	0.14	0.39	−11.99	Increase	6.14	
	2	58218738	rs6742445	A	T	0.02	0.07	0.4	−4.83	Increase	3.28	
	3	30504455	rs72847487	C	T	0.06	0.13	0.33	−7.28	Increase	2.20	
	3	45527650	rs2578673	C	T	0.02	0.11	0.4	−8.62	Decrease	5.07	
	3	65852193	rs77700841	G	C	0.04	0.12	0.34	−7.33	Increase	2.75	
	4	9686390	rs7691759	C	T	0.18	0.40	0.21	−21.49	Decrease	2.17	
	4	40240871	rs148332376	C	T	0.03	0.09	0.4	−6.28	Increase	2.96	
	4	99522696	rs17029090	A	G	0.03	0.12	0.35	−9.19	Increase	4.32	
	5	179974542	rs17079875	G	A	0.02	0.07	0.37	−4.57	Increase	2.86	
	6	31368014	rs57989216	G	A	0.02	0.04	0.4	−2.13	Increase	2.25	
	6	33957119	rs9688730	A	C	0.03	0.07	0.42	−3.76	Increase	2.10	
	6	144158325	rs7755842	A	T	0.03	0.14	0.36	−11.31	Increase	5.21	
	6	148588135	rs60463810	C	T	0.05	0.11	0.3	−6.51	Increase	2.36	
	7	151202976	rs61733346	C	G	0.04	0.10	0.33	−6.06	Increase	2.62	
	8	54303623	rs56775982	C	T	0.05	0.14	0.34	−9.02	Increase	2.78	
	10	76449748	rs72642263	A	G	0.05	0.14	0.33	−8.24	Increase	2.51	
	11	46918742	rs78245588	C	T	0.02	0.09	0.44	−6.95	Increase	5.30	
	11	129980320	rs7130078	G	A	0.02	0.06	0.44	−3.60	Increase	2.83	
	11	130190674	rs7126904	G	A	0.03	0.09	0.44	−6.44	Increase	3.45	
	12	111266635	rs2157999	G	A	0.04	0.15	0.35	−10.89	Increase	3.66	
	13	41459562	rs10467472	G	C	0.11	0.22	0.22	−11.03	Increase	2.00	
	16	3739076	rs3025678	C	T	0.02	0.07	0.43	−5.32	Increase	3.63	
	18	5940342	rs8094014	A	G	0.08	0.28	0.3	−19.60	Increase	3.35	
	18	48131482	rs79272944	G	T	0.02	0.08	0.4	−5.28	Increase	3.21	
	19	8085509	rs17160151	C	T	0.02	0.07	0.42	−4.74	Increase	3.12	
	19	54162804	rs9636146	G	A	0.04	0.10	0.4	−5.45	Increase	2.25	
	21	26678731	rs9975780	C	T	0.03	0.12	0.33	−8.90	Increase	4.07	
	21	43056923	rs1005585	T	C	0.07	0.15	0.29	−7.88	Increase	2.18	
	21	46430193	rs8131693	G	A	0.02	0.06	0.43	−4.58	Increase	3.57	

CHR: chromosome number; POS: genomic position; REF: reference allele; ALT: alternative allele; ALT_AF: alternative allele frequency; OR: odds ratios; BETA, beta coefficient; FC: fold change in AF.

## Data Availability

Datasets analyzed in this study were obtained from Qatar Biobank (accessed on 9 June 2020) and are subject to data access and sharing policies due to privacy and ethical restrictions. Access to the raw data can be requested from Qatar Precision Health Institute (QPHI) following their application procedures at https://www.qphi.org.qa/research/how-to-apply (accessed on 9 June 2020). The summarized and derived data supporting the findings of this study are presented within the article and are also available from the corresponding author upon reasonable request.
